# Chemotherapy Regimens for Non-Metastatic Conventional Appendicular Osteosarcoma: A Literature Review Based on the Outcomes

**DOI:** 10.3390/curroncol30070457

**Published:** 2023-06-27

**Authors:** Joaquim Soares do Brito, Rodrigo Santos, Marco Sarmento, Pedro Fernandes, José Portela

**Affiliations:** 1 Centro Hospitalar Universitário Lisboa Norte, 1649-035 Lisbon, Portugal; pmfernandes@medicina.ulisboa.pt (P.F.); jose.portela@jmellosaude.pt (J.P.); 2 Faculdade de Medicina da Universidade de Lisboa, 1649-035 Lisbon, Portugal; rmrsantos@campus.ul.pt; 3 Hospital CUF Descobertas, 1998-018 Lisbon, Portugal; marco.sarmento@sapo.pt

**Keywords:** neoadjuvant chemotherapy, adjuvant chemotherapy, osteosarcoma, outcomes

## Abstract

Osteosarcoma is a rare condition with a complex treatment. Most protocols include neoadjuvant chemotherapy, surgery, and consolidation chemotherapy as the standard of treatment. However, the use of neoadjuvant chemotherapy lacks scientific evidence proving superiority in opposition to the use of isolated chemotherapy in an adjuvant regimen after surgery. We conducted a review for studies published in English between 1980 and 2020, using the MEDLINE/PubMed and Scopus electronic databases, to evaluate the outcomes when using neoadjuvant chemotherapy or adjuvant chemotherapy strategies in the treatment of non-metastatic appendicular osteosarcoma, as well as the toxicity associated with different chemotherapeutic regimens. Patients were divided into a neoadjuvant chemotherapy group (NAC) and adjuvant chemotherapy group (ACT), depending on the chemotherapy regimen used in association with surgery. A total of 1254 articles in English were screened by title and abstract, and 146 were pre-selected for full reading and analysis. A total of 24 assays matching the inclusion criteria were selected: 10 prospective and 14 retrospective studies. This review points to an absence of significative differences in outcomes, namely overall survival, disease-free survival/event-free survival rates, or toxicity, regarding neoadjuvant or single adjuvant chemotherapy strategies used in the treatment of appendicular non-metastatic osteosarcomas. However, there is a significative difference in population dimensions between the NAC and the ACT groups. Additionally, clinical presentation, tumor localization, tumor volume, or histological type were not considered, with these variables presenting the potential to influence these results. Despite these limitations, our findings should allow a re-thinking of our current practice and promote new opportunities to optimize treatment, always looking towards better survival and lower complications rates.

## 1. Introduction

Osteosarcoma is the most frequent primary malignant bone tumor; it is the third most common tumor in adolescence, following lymphomas and brain tumors [1]. Nonetheless, it is a rare disease with an incidence in Europe of 0.2–0.3/100.000 cases per year among the general population and 0.8–11/100.000 per year in those between 15 and 19 years old, with a slight male predominance (1.4:1) [2,3,4]. Osteosarcoma is histologically characterized by excessive proliferation of abnormal osteoid producing mesenchymal cells, which can affect any bone [5]. The most affected anatomical region is the metaphysis of long bones, namely in the distal femur, proximal tibia, and proximal humerus, with more than half of cases developing around the knee joint [1,5]. Nearly 10% of osteosarcomas can arise in the axial skeleton, predominantly involving the pelvis. This axial involvement is more frequent in older patients [1]. Surgery remains the basilar treatment for osteosarcoma. There has been a transition from amputation to limb-salvage surgery, with biological reconstruction, megaprostheses, and a combination of both or other complex procedures such as rotationplasties, as the mainstay in current practice [6]. Despite the relevance of surgery, the absence of chemotherapy is related to extremely unfavorable outcomes. Past experiences demonstrate that nearly 90% of patients on whom isolated surgery was performed died due to pulmonary metastasis within 12 months. This was associated with the concept of micrometastatic spread, which was probably already present at the time of surgery [1,5,7]. In this setting, Marcove et al. reported on 145 consecutive patients with a 5-year disease-free survival rate of 17.4%, with six months mediating between the presence of metastasis and death [8]. The use of chemotherapy to treat osteosarcomas emerged between 1970 and 1980. Several reports from different institutions showed a significant improvement in the disease-free interval and in the overall survival, surpassing 50%. Nonetheless, it was not clear whether these apparently improved outcomes were either due to chemotherapy or to a change in the natural history of the disease, improved staging procedures, refinement of the pathologic definition of classic osteosarcoma, or more aggressive approaches towards pulmonary metastasis [7]. In addition, none of the studies that aimed to prove the efficacy of chemotherapy had a randomized control group. The efficacy of chemotherapy was mainly proved by two North American randomized studies [7,9]. These studies allowed a direct comparison between the use of adjuvant chemotherapy and its absence, demonstrating a clear benefit in the event-free survival and in the overall survival. Compared to surgical treatment alone, chemotherapy increased the likelihood of disease-free survival from 10–20% to around 60% at a median follow-up of five years [3]. This chemotherapy was mainly based on doxorubicin (ADM), cisplatin (CIS), high-dose methotrexate (HDMTX) with rescue leukovorin, and ifosfamide (IFO). However, the best combination remains to be defined [10]. In most protocols, the use of neoadjuvant chemotherapy strategy (NAC), which means doing several chemotherapy cycles before surgery, prevails, usually being followed by more chemotherapy after the surgical procedure. Nonetheless, there is no scientific evidence that NAC provides greater benefits in terms of survival compared to the use of chemotherapy in adjuvancy (ACT), which implies chemotherapy after the surgical treatment is done [5,10]. Despite the regimen including NAC followed by surgery and consolidation chemotherapy being the most popular, there is poor evidence proving the superiority of this strategy towards ACT. With this manuscript, we intended to compare the literature regarding these two different protocols to treat appendicular bone osteosarcoma. In our conclusion, we hope to clarify the effect obtained with each one of these therapeutic regimens and to propose the most valuable option to achieve success in bone osteosarcoma treatment (Table 1).

## 2. Materials and Methods

This study is a review of previously published studies in the literature in English concerning the outcomes with the use of a neoadjuvant chemotherapy regimen in addition to surgery, and chemotherapy in an adjuvant setting (only after surgery) for the treatment of appendicular non-metastatic osteosarcoma. Two electronic databases were used: MEDLINE/PubMed and Scopus databases, searching between 1980 and December 2020. We searched for studies that included the keywords: “Neoadjuvant Chemotherapy” OR “Adjuvant Chemotherapy (MeSH)” AND “Osteosarcoma (MeSH)” AND “Outcome”. The last date of search was 19 January 2021. For the inclusion criteria, we applied the Population, Intervention, Comparison, Outcome, and Study strategy. We defined:

1. Population: patients with primary non-metastatic conventional localized appendicular osteosarcoma. Regarding age, in this systematic review we excluded articles referring only to patients >40 years old or <10 years old.
2. Intervention: treatment using neoadjuvant chemotherapy, surgery, and consolidation chemotherapy, which we compared to treatment with surgery and adjuvant chemotherapy only.
3. Outcomes: (a) primary outcomes: overall survival, disease-free survival, event-free survival; (b) secondary outcomes: toxicity.
4. For this type of study, we included prospective and retrospective observational studies, randomized controlled trials, case-controlled studies, and cohort studies.

For the exclusion criteria we considered the following: review articles, case report studies, articles with only an abstract available, and articles where the full text was not accessible.

## 3. Results

### 3.1. Selected Studies

**Figure 1:**
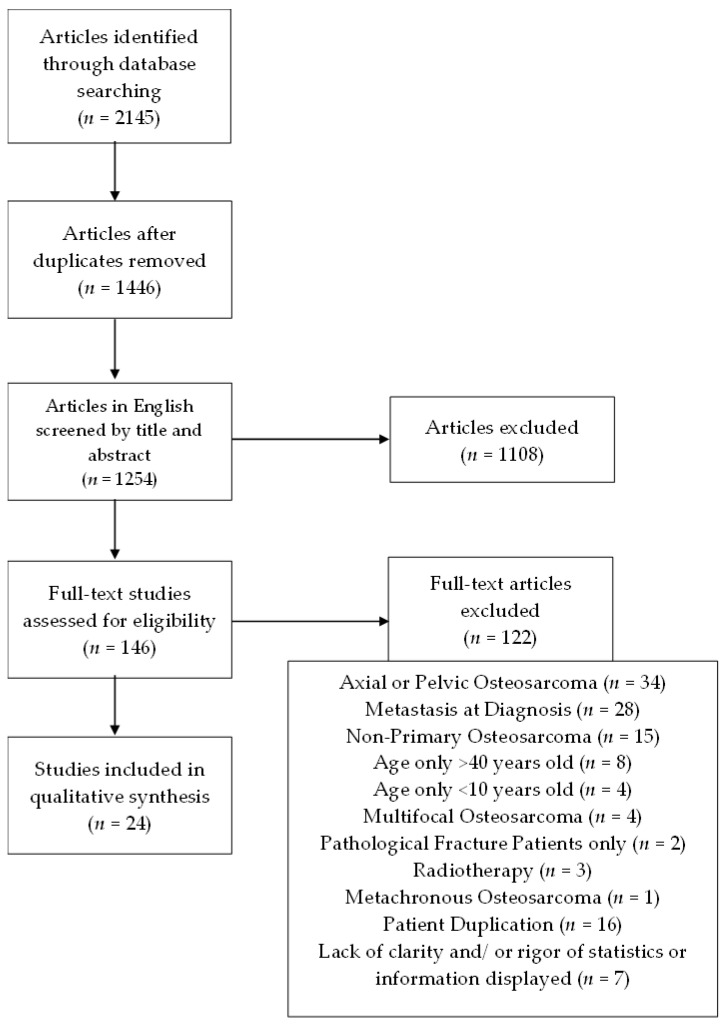
Flowchart illustrating the search strategy and number of records screened and included.

A total of 2145 articles (990 from MEDLINE/PubMed and 1155 from Scopus databases) were initially obtained with the keywords used. We then proceeded to remove the 699 duplicated articles, ending up with a total of 1446 articles, from which only 1254 were in English. Out of those 1254 articles, we removed 1108 according to our previously defined inclusion and exclusion criteria. The selection out of the 146 remaining articles underwent an integral reading, a rigorous analysis, and confirmation of the keywords searched to meet the inclusion and exclusion criteria. From the 146 articles screened we removed: 34 due to presence of axial or pelvic osteosarcoma; 28 due to presence of metastasis at the time of diagnosis; 15 concerning only recurrent osteosarcoma; eight regarding only people above forty years of age; four regarding only children less than 10 years of age; two due to the exclusive presence of patients presenting pathological fracture at the time of diagnosis; one due to the presence of metachronous osteosarcoma; four due to the presence of multifocal osteosarcoma; and three due to the use of radiotherapy, which is not included in either of the comparison arms. We removed 16 articles from this review due to duplication of patients; some articles included patients previously included in articles from the same institution. We excluded seven more articles due to lack of clarity and rigor of the statistics and/or information presented. Therefore, 24 articles were selected for analysis (Figure 1).

### 3.2. Primary Outcomes

#### Overall Survival (OS), Disease-Free Survival (DFS)/Event-Free Survival (EFS)

Ettinger et al. followed the ACT strategy, using an adjuvant chemotherapeutic regimen consisting of ADM and CIS. The 72-month overall survival reported was 77% and the estimate 72-month disease-free survival was 64%. Local and metastatic pulmonary recurrences were reported in two and six patients, respectively [13], as shown in Table 2. In Glasser et al., 240 patients followed the NAC strategy, while the remaining 39 followed the ACT strategy. In this study, four different NAC protocols were used: HDMTX with leucovorin rescue, a combination of bleomycin, cyclophosphamide, or dactinomycin (BCD), and ADM. The postoperative chemotherapy was modified according to the histological response of the primary tumor [14]. The OS for those that underwent single adjuvant chemotherapy was 77% at 5 and 10 years, while for those in the NAC group it was 78% at 5 and 74% at 10 years, respectively [14]. Both groups showed a similar EFS. Smeland et al. used HDMTX, CIS, and ADM as neoadjuvant chemotherapy regimens, adding ETO and IFO to poor responders. The projected survival at 5 years was 74%. They outlined an EFS at 5 years of 61% [15]. Goorin et al. compared randomly assigned patients to the NAC group (45 patients) and to the ACT group (55 patients) [10]. Chemotherapy included HDMTX with leucovorin rescue, ADM, CIS, and BCD, with treatment arms presenting similar incidences of limb salvage surgery. The OS for the NAC and ACT groups at 5 years was 76% and 79%, respectively. They reported that twenty-eight patients in the NAC group remained disease-free at the time of report, eight had distant relapses and six had locally progressive disease while receiving presurgical chemotherapy, two of which also developed lung metastases. Among the ACT group, 39 remained disease-free and 15 developed distant metastases. The 5-year EFS for the NAC and ACT groups was 61% and 69%, respectively [10]. Zalupski et al. used a combination of ADM, CIS, and IFO as chemotherapeutic regimens in a preoperative and postoperative setting (NAC strategy). The 5-year OS was 58% and the median OS was 86 months [16]. Ford et al. compared two centers. A total of nine different chemotherapy regimens were used, but all of them used the NAC strategy. Center one included three- to four-drug regimens (ADM + CIS + MTX and IFO) and Center two used a two-drug regimen (ADM + CIS). The 5-year OS was 73% at Center one and 60% at Center two. A 5-year DFS of 56% in Center one and 43% in Center two were also reported [17].

Wilkins et al. followed the NAC strategy. These authors selected 205 intravenous ADM and intra-arterial CIS as drugs for chemotherapy. At 5 and 10 years, the OS was 96.6% and 93.2%, respectively. The EFS was 86.4% at 5 and 10 years. In this study, the authors tailored the postsurgical chemotherapy according to neoadjuvant therapy, individualizing treatment based on arteriographic response. To these authors, tailoring chemotherapy according to arteriographic response improves the chances of achieving a good histologic response and avoids unnecessary toxicity for rapid responders [18]. Bacci et al. retrospectively analyzed 1148 patients treated in a single institution from 1972 to 1999, with four different protocols of adjuvant-only chemotherapy (ACT) and seven different protocols of neoadjuvant chemotherapy (NAC). All patients were treated at the same institution by the same team, and they were followed for a minimum of five years unless they relapsed or died. The 5-year OS was 66% for the entire cohort and the 10-year OS was 57%. The entire cohort EFS was 57% at 5 years. The 5-year EFS for the adjuvant protocols group was 43%. Meanwhile, those who went through neoadjuvant protocols had a 5-year EFS of 61%. According to this retrospective study, there were no significant differences in 5-year EFS between patients treated with different protocols. The average time to relapse was longer in neoadjuvant group than in patients of adjuvant group [19]. Ferrari et al. (2005) followed an NAC strategy based on high-dose IFO, MTX, CIS, and ADM. The 5-year overall survival was 77% and the 5-year EFS was 64%. With a median follow-up of 55 months, 119 patients were event free; 59 had local recurrence or distant relapse, three died due to chemotherapy-related toxicity, and one died because of pulmonary embolism. The local recurrence rate was 4% [20]. Petrilli et al. also followed the NAC strategy and promoted two studies in their analysis. One study used epirubicin (EPI), carboplatin (CBP), IFO, and HDMTX. The other used CBP, CIS, ADM, and IFO. The estimated 5- and 10-year survival rates for the non-metastatic patients (168 out of 209 included in this study) were 60.5% and 55.2%, respectively. They reported an EFS rate of 45.5% at both 5 and 10 years for the same subgroup of patients [21]. Basaran et al. used EPI, CIS, and IFO in an NAC setting. The 5-year OS was 48.2% and the 5-year EFS was 41.9% [22]. Lewis et al. had patients assigned to two different NAC regimens: Regimen-C (conventional) and Regimen-DI (dose-intensive). Both used only CIS and ADM in neoadjuvant and adjuvant settings with an overall equivalent chemotherapeutic dosage. Nonetheless, Regimen-DI consisted in a shorter treatment timespan. From a total of 497 patients, 245 and 252 patients were analyzed for Regimen-C and Regimen-DI, respectively. The 5-year OS was 55% for Regimen-C and 58% for Regimen-DI. The authors came to an estimated 5-year progression-free survival of 39% for Regimen-C and 41% for Regimen-DI [23]. Iwamoto et al. used HDMX, CIS, and ADM as preoperative chemotherapy with IFO addition if the induction therapy was assessed as not effective. The 5-year OS was 77.9%. They also reported an EFS of 65.5% at 5 years [24].

Bielack et al. advocate for the NAC strategy. The results presented are from a large, multicentric and multidisciplinary osteosarcoma treatment network. Most protocols included neoadjuvant chemotherapy with HDMTX, ADM, CIS, and IFO. Five- and 10-year OS estimates were 74.8% and 69.5% for the 2017 patients included in this study [25]. Hong et al. enrolled 124 patients in two NAC groups: the AP group (77 patients) received a double regimen of intra-arterial CIS and intravenous ADM. The IAP group (47 patients) received a triple regimen of additional intravenous IFO. The 5-year OS rate was 71% (85% in the AP group and 63.9% in the IAP group). The authors outlined a 5-year DFS rate of 66.5% (78.7% in the AP group and 63.6% in the IAP group) [26]. Smeland et al. (2011) also followed an NAC strategy using MTX, CIS, and ADM, with IFO used for poor histological responders. The 5-year OS was 76% and the estimated event-free survival at 5 years was 65%. The median time from diagnosis to relapse was 20 months [27]. Li et al. distinguished two NAC groups: 24 patients that only received two cycles of EPI and CIS (nonstandard chemotherapy group) and 87 patients that received three to six cycles of the same induction chemotherapy (standard chemotherapy group). The 3-year OS for the entire cohort was 68.3%, however, there were significant differences in the OS rate between the nonstandard and standard chemotherapy group (38.9% vs. 80.0%). The authors reported a 3- year DFS of 65.9% in the entire cohort, also with significant differences between DFS of nonstandard and standard chemotherapy groups (30.1% vs. 79.5%) [28]. Whelan et al. used data from three randomized controlled studies (1067 patients) where the standard treatment was ADM and CIS, following the NAC strategy. The 5- and 10-year OS was 56% and 52%, respectively. Progression-free survival (defined as DFS or EFS in other studies) at 5 and 10 years was 43% and 42%, respectively [29]. Kudawara et al. followed the NAC strategy using ADM and CIS, with IFO and HDMTX in addition during adjuvant chemotherapy. The 5- and 10-year OS rate was 98% and 95%, respectively. The EFS rate at 5 and 10 years was 83 and 80%, respectively. Eight patients developed pulmonary metastasis and one patient developed bone metastasis 24 months after the initial treatment [30]. Xu et al. used a neoadjuvant chemotherapy regimen consisting of CIS, IFO, and ADM. The estimated 5-year rate of OS was 61.5%. The EFS rate estimated at 5 years was 54.8% [31].

Ferrari et al. (2014) used an NAC strategy consisting of a MAP regimen (273 MTX, ADM, CIS) with the addition of IFO in poor responder patients (MAPI regimen). The 5-year OS rate was 81%. They described a 5-year EFS rate of 50%. Overall, from 171 patients, 103 received all drugs in the protocol without any dose reduction. In this subgroup, a trend towards a better EFS at 5 years was observed in 69 patients whose chemotherapy duration was longer than planned according to protocol [32]. For Bajpai et al., the NAC was based on ADM, CIS, and IFO. Of 237 non-metastatic patients, only 217 completed neoadjuvant chemotherapy and surgery. From these, only 209 completed the intended adjuvant chemotherapy. Estimated 3- and 5-year OS for those who completed treatment was 82% and 80%, respectively. At the median follow-up, there were 32 total deaths: 31 due to progressive disease and one due to secondary acute leukemia. Bajpai et al. showed an estimated 3-year and 5-year EFS of 63% and 60%, respectively, for those 209 patients who followed the treatment protocol [33]. In the study promoted by Huang et al., neoadjuvant chemotherapy consisted of IFO, ADM, and MTX/CIS. The 3- and 5-year OS rates were 91.3% and 87%, respectively [34]. Morsy et al. used ADM and CIS as neoadjuvant chemotherapy. Estimates for OS at 3 and 5 years was 79% and 65.3%, respectively. The EFS rate at 3 and 5 years was 69.5% and 65.2%, respectively. Of 30 patients, 20 remained continuously disease free, and from 10 patients who experienced events, isolated distant metastases occurred in eight, combined distant and local metastases occurred in one patient, and one patient relapsed locally. The median time to recurrence was 25 months [35].

### 3.3. Secondary Outcomes

#### Treatment-Related Toxicity

Ettinger et al. did not report treatment-related deaths. A single patient developed congestive heart failure and required treatment with digitalics. Nephrotoxicity was seen in one third of patients, and there was loss of hearing and speech frequency, not always completely reversible [13]. Smeland et al. reported toxicity due to HDMTX in nine patients, CIS in three, and one case from unknown causes. These 13 patients had their chemotherapy modified or terminated. There were three treatment-related deaths [15]. Goorin et al. reported two deaths from congestive heart failure (anthracycline cardiomyopathy) in the NAC group and, in the other case, due to medulloblastoma. In the ACT group, there was one toxic death from bronchiolitis obliterans, which was most likely caused by bleomycin toxicity. One patient had significant hearing loss but the group he belonged to is unknown [10].

Zalupski et al. reported one death due to infection associated with severe myelosuppression. In addition, two pulmonary metastatic nodules were found in the same patient. Hematologic toxicities were common with blood transfusions, which were required in 34 patients. Platelet transfusions were required in six patients per chemotherapy period. Toxicities led to a premature termination in at least 11 patients [16]. Wilkins et al. did not report toxic deaths. One patient developed severe cardiotoxicity and another severe ototoxicity [18]. Bacci et al. reported 18 deaths related to chemotherapy toxicity. Of these, 10 were due to doxorubicin-induced cardiotoxicity; six from sepsis due to persistent leukopenia after CIS/ADM cycles; one from renal failure due to MTX; and one from hepatic veno-occlusive disease after MTX. Six of these patients received adjuvant and 12 were treated with neoadjuvant chemotherapy. Fifteen other patients experienced severe cardiotoxicity. There were also neurologic disturbances (12 cases) after ifosfamide and CIS-related neuropathy or ototoxicity (24 cases). Second neoplasms were also identified: acute lymphoblastic leukemia in four cases; chronic myeloid leukemia in two; lung cancer in three; breast cancer in five; CNS tumors in two; soft tissue sarcomas in two cases; and renal cell carcinoma in one [19]. Ferrari et al. outlined three deaths due to sepsis and acute renal failure. Two patients died during the preoperative phase and the third during postoperative chemotherapy. Renal failure occurred in six patients preoperatively and in 10 postoperatively. Acute heart failure developed in one patient four months after chemotherapy. Hearing loss was found in 40% of patients. Azoospermia was recorded in 15 cases (the entire group that underwent seminal analysis). Permanent amenorrhea was recorded in two patients, both 40 years old. Regarding hematologic toxicity, all patients experienced severe platelet and leukocyte toxicity [20].

Basaran et al. outlined the frequent hematologic toxicity, with neutropenia being the most prominent life-threatening toxicity. Febrile neutropenia was reported in nine patients (24%). In addition, 21 patients experienced reversible grade three non-hematological toxicity. No severe or life-threatening bladder, ototoxicity, or acute cardiac toxicity were observed. There were no treatment-related deaths in this study [22]. Lewis et al. reported toxicity in 12 patients included in the Regimen-341 C (5%) and 17 patients from those in Regimen-DI group (7%). According to this study, 24 (10%) and 25 (10%) patients in Regimen-C and Regimen-DI, respectively, stopped chemotherapy prematurely due to excessive toxicity or patient choice. Three deaths occurred during the protocol treatment period, all among patients allocated to Regimen-DI. Nearly all the patients (468/97%) experienced severe toxicity [23]. Iwamoto et al. observed three deaths due to cardiomyopathy induced by ADM, one due to infection, one due to secondary leukemia, and another classified as direct treatment toxicity. Severe and life-threatening hematologic toxicity was frequent and severe/life-threatening non-hematologic toxicities were found in 22.4% of the cycles [24]. Bielack et al. reported 37 deaths during primary treatment in the entire cohort, however, did not differentiate between metastatic and non-metastatic patients. Myelotoxicity was the cause on 27 occasions, and cardiac failure in two cases [25]. Hong et al. reported more hematologic toxicity in the IAP group. Some severe and life threatening non-hematologic toxicities were also reported. There were no treatment-related deaths during neoadjuvant chemotherapy, however, five patients died from treatment-related septic shock during adjuvant chemotherapy (two in the AP group and three in the IAP group) [26]. Smeland et al. (2011) reported three toxic deaths. In addition, two cases of life-threatening toxicity were observed [27]. Li et al. reported that major complication was neutropenia, however, there is no reference to treatment-related deaths [26]. Whelan et al. reported early termination of chemotherapy in 148 patients either due to toxicity or patient refusal [29]. Kudawara et al. outlined interruption of IFO due to two episodes of neurotoxicity and six episodes of life-threatening neutropenia. There were no treatment-related deaths but there was one case of life-threatening nephrotoxicity. No late complications such as cardiotoxicity or second cancer were reported [30]. Xu et al. reported few serious side effects, and the most common events, myelosuppression, nausea, vomiting, abnormal liver function, and alopecia, were transient [31]. In a study by Ferrari et al. (2014), no treatment-related deaths were recorded. However, all patients experienced one or more episodes of life-threatening leukopenia [32]. Bajpai et al. reported that 42 patients (18%) required dose reductions during chemotherapy owing to toxicity. There were two chemotoxic reported deaths [33] as shown in Table 3.

## 4. Discussion

Despite being the most frequent malignant bone tumor, osteosarcoma is a rare disease, developing most commonly in the metaphysis of long bones [1,4,5]. Most osteosarcomas have an unknown origin, although there are recognized risk factors, such as previous radiotherapy, Paget’s disease, and germline genetic abnormalities, associated with some syndromes [36,37,38]. There are several histologic types of osteosarcomas, but the central conventional type is by far the most prevalent [39]. Osteosarcoma typically presents with localized non-mechanical pain, swelling, and limited joint movement, although the first sign can also be a pathological fracture [5]. These signs and symptoms should prompt a radiological evaluation, and differential diagnosis will include osteomyelitis, benign tumors, or metastases, any of which is more common than osteosarcoma. However, age is an important factor and aids proper diagnosis, since osteosarcoma is infrequent in children below five or in adults above 40 years of age [38,39]. In the osteosarcoma setting, tumor volume assessment, staging, and confirmative biopsy are paramount [3]. Treatment of osteosarcoma has always been challenging. Despite slight changes in protocols based on pharmacological changes and/or drug doses, no significant improvement has been noted in patients’ survival in the last 30 years [40]. Nonetheless, the advent of chemotherapy drastically improved the outcome for osteosarcoma patients, who, with surgery-based treatments, would die due to subclinical and undetectable micrometastasis [1,5]. As such, therapeutic regimens with neoadjuvant chemotherapy followed by surgical tumor resection and subsequent adjuvant chemotherapy became gold standard, leading to success in almost two thirds of the patients with localized disease [41]. However, the shift from single adjuvant chemotherapy towards neoadjuvant chemotherapy was mostly based on non-scientific premises: it can cap the inflammatory response in a tumor’s periphery; inhibit neovascularization; decrease tumor size, allowing an easier resection; it allows prompt administration of systemic therapy treating microscopic disease; offers time for surgical planning (including manufacture of custom-made megaprosthesis); allows for the evaluation of the chemotherapy’s histological effect; and permits the subsequent tailoring of consolidation therapy [18,19,27,41,42]. Despite all of these principles developed during the early stages of osteosarcoma multidisciplinary treatment, none of them was rationally proved. Instead, the higher number of limb salvages performed in patients who receive NAC seems to relate more to early tumor detection and having a higher number of reconstructive solutions, rather than to the reduction of the tumor mass by preoperative treatment [19]. Moreover, it has been demonstrated that adding IFO and ETO to the backbone of treatment (HDMTX, ADM, CIS) for patients with poor response to preoperative treatment resulted in similar event-free survival, with increased toxicity and more secondary malignancies [43]. Simultaneously, the advent of modular megaprothesis, the development of bone banks, and improved skills in biologic reconstructive techniques using bone and soft-tissue-free flaps, virtually eliminated the need for time-consuming custom-made megaprosthesis manufacture [44]. Therefore, despite the widespread acceptance of neoadjuvant chemotherapy in the treatment of osteosarcoma, there is no proof of its superiority when compared with chemotherapy used solely in an adjuvant setting regarding survival and disease/event-free survival. Additionally, it would be interesting to understand the weight of toxic side adverse effects in patients going through NAC or ACT strategies. This review found four papers (including 364 patients) under ACT to treat appendicular osteosarcomas [10,13,14,19]. In the study promoted by Glasser et al., the ACT group was represented by those patients with pathologic fractures, infected biopsy sites or fungating tumors. This is a critical aspect, since it might negatively affect the final prognosis for these patients [14]. Bacci et al. analyzed 248 patients treated with ACT protocols and 900 that went through NAC protocols between 1972 and 1999 in the same institution. Although this nonrandomized study showed improvement in the outcomes, including more recent NAC protocols, nonetheless there was a long time period of patient recruitment, which meant simultaneous improvement of pharmacological, radiological, and surgical techniques to treat those patients where NAC was used [19]. Despite the use of preoperative chemotherapy in most protocols, Goorin et al. questioned the superiority of neoadjuvant chemotherapy and promoted a direct comparison between NAC and ACT. In this study, the superiority of neoadjuvant chemotherapy was not proven [10]. Nonetheless, and as these authors pointed out, a more representative population would be needed to strengthen this conclusion. The 5-year OS rate of patients treated with adjuvant chemotherapy, considering the available data by Ettinger et al., Glasser et al., and Goorin et al., varies from 77% to 79%. Bacci et al. do not present the difference in OS between the ACT group and the NAC group. The 5-year DFS/EFS reported on the same studies varies from 43% to 69%, with Glasser et al. reporting only the DFS/EFS for the entire cohort [10,13,14,19]. Most studies included in this review used the NAC strategy. Basaran et al. reported a lower 5-year OS (48.2%); however, these results may have been influenced by higher patient age and tumor size (55% of patients presented with tumors larger than 10 cm). Wilkins et al. and Kudawara et al. presented >95% OS at 5 years, but we must stress that these results are obtained from small sample sizes and from particularly young populations. In general, we observed that the 5-year OS rate had a wide range depending on the studies (Table 3).

Bacci et al., Bielack et al., and Whelan et al.’s retrospective studies had the biggest sample sizes in this review. Bacci et al. presents the only study that reports the results based on a single institution. The 5-year OS rate was 66%, but this number concerns all patients, even though the majority underwent NAC protocols. The major weak points for these results are related to the 28-year recruiting period and the nonrandomized nature of the series. During the span of the study, new drugs, new imaging modalities, and new surgical techniques were introduced, and these technological advances must be considered when analyzing the results. Whelan et al. used ADM and CIS as neoadjuvant regimens. The use of only two agents might have been the reason why the 5-year OS presented by these authors (56%) is lower than most studies used in this review. Bielack et al. reported the results from a large, multicentric network, using neoadjuvant protocols based on HDMTX with several combinations of CIS, ADM, IFO, and CBP/ETO/BCD, additionally. The authors showed a 5-year OS rate of 74.8% for the patients with non-metastatic appendicular osteosarcoma. 

Overall, the 5-year OS rate between the ACT and the NAC group seem similar, despite the discrepancy among sample sizes (Table 3). The major limitation inherent to this review is precisely the very small sample that represents the ACT group. Furthermore, the OS of 10 years is not referred to in ACT studies and is addressed only in a few NAC groups. The DFS and EFS at five years presents a wide range, from 39% to 65.5% in the NAC group. In the ACT group we found DFS/EFS at 5 years of 43%, 64%, and 69.8%. Once again, and despite the difference in representation of patients in each group, we can observe a similar range. This may suggest or at least glance at the possibility that the use of neoadjuvant chemotherapy may not be superior to simple adjuvant chemotherapy. Regarding the toxic adverse effects caused by chemotherapy in the ACT group [10,20,26], the most important side effect recorded was cardiotoxicity related to ADM. There was one death related to bronchiolitis obliterans, but it was most likely caused by bleomycin, which is not currently part of the four most active agents currently used. Bacci et al. also reported six deaths from sepsis during persistent leukopenia after CIS/ADM cycles, one death due to renal failure related to MTX and one death related to hepatic veno-occlusive disease after a cycle of MTX. These findings cannot be directly attributed to either of the study groups, since it is not explicit as to which group the patients belong. Nonetheless, there were 18 deaths reported related to treatment toxicity, six in the ACT group (6/248) and 12 in the NAC group (12/900). Nephrotoxicity, ototoxicity with hearing loss, neurologic disturbances, and even secondary neoplasms were also noted.

Regarding the studies including NAC patients, early termination/modification of chemotherapy was frequently reported due to adverse toxic effects (125 patients) [15,16,23]. Hematologic toxicity was very commonly reported in all studies included in the review. Other toxicities were also reported, namely severe cardiotoxicity [16], severe ototoxicity [18], azoospermia [20], amenorrhea [20], gastrointestinal disorders, electrolyte abnormalities, neurological toxicity, and infections [24]. The most serious adverse toxic effect reported was death. There were at least 25 treatment-related deaths [15,16,20,24,33] due to diverse causes: cardiac arrest associated with cardiomyopathy induced by ADM [15,24], leukemia [15,24], acute respiratory distress syndrome [13], and infection/sepsis post-myelosuppression [13,16,18,24,26,33] were the most common causes.

As observed in Table 3, we can see that reported deaths directly attributed to chemotherapy range from 0.37% (NAC) to 1.92% (ACT). Based on this, we could advocate for the NAC strategy as less toxic than the ACT strategy. However, additionally to the existing difference in population dimensions, we must stress that the ACT strategy was used in older studies. As such, a better monitoring and responding capacity towards toxic events is expected in the most recent studies, influencing chemotherapy-inherent toxicity-related outcomes. It is the authors’ understanding that this review brings new light regarding the role of NAC and ACT regimens in appendicular osteosarcomas. Despite huge discrepancies regarding group sizes (364 patients in the ACT group and 6789 patients in the NAC group), this review could not find significant differences in the OS or DFS/EFS using NAC or ACT strategies in the treatment of appendicular non-metastatic osteosarcoma. Nonetheless, all variables that can influence these outcomes on an individual scale, such as clinical presentation, tumor localization, tumor volume, or histological type, were not considered. Individual features and intrinsic treatment characteristics, such as the drugs used and dosages, type of surgery performed, and even the institution where the treatment took place, were not considered. In addition, we must also stress that there is not a common definition of the concepts of OS, DFS, and EFS among studies, which also can affect the results.

## 5. Conclusions

Chemotherapy in a neoadjuvant setting to treat osteosarcoma was begun based on precedents set in the last century and it has prevailed on most protocols since then. However, its superiority towards the use of chemotherapy only in an adjuvant setting remains to be proven. Despite obvious limitations, this review showed that survival does not seem to differ among NAC or ACT strategies. This finding should allow us to criticize our current practice and promote new opportunities to optimize appendicular osteosarcoma treatment, always looking towards better survival and lower complication rates.

## Figures and Tables

**Table 1:** Summary of therapeutic options for the management of osteosarcoma [11,12].

Therapeutic Modality	Therapeutic Relevance *	Comments
Chemotherapy	++++	Relevant toxic and side effects (cardiotoxicity, liver and kidney toxicity, bone marrow suppression, neurotoxicity.) Cancer resistance to chemotherapeutics.
Surgery	++++	Only allows for local control of the disease.
Radiotherapy	++	Some impact in unresectable osteosarcomas, in those osteosarcomas where tumor remains on the resection margin and for patients with poor response to chemotherapy.
Immunotherapy	++	Osteosarcoma cells have low immunogenicity and, hence, therapeutic response is limited.
Embolization	+++	Relevant only for pain control; cannot control local or systemic disease.

* + Not relevant; ++ scarcely relevant; +++ relevant; ++++ highly relevant.

**Table 2:** Characteristics of the studies included and summary of the results.

Author	Year	Type of Study	No. of Patients	Age Median Years (Range)	Average Follow-Up (Months)	Most Common Tumor Location	Treatment	OS	DFS/EFS	TR Toxicity (Major Events)
Ettinger et al. [13]	1986	Prospective	22	17 (6–46)	70	DF (*n* = 12) PT (*n* = 6)	ACT (*n* = 22)	72 m: 77%	72 m: 64%	Deaths (*n* = 0)
Glasser et al. [14]	1991	Retrospective	279	Male: 17 (5–63) Female: 15 (3–58)	92	DF (*n* = 131) PH (*n* = 52) PT (*n* = 52) PF (*n* = 14)	NAC (*n* = 240)	5 y: 78% * 10 y: 74% *	5 y: 70% 10 y: 69%	NR
							ACT (*n* = 39)	5 y: 77% * 10 y: 77% *		
Smeland et al. [15]	2003	Retrospective	113	<15 (*n* = 34) >15 (*n* = 79)	83	Femur (*n* = 60) Tibia (*n* = 31) Humerus (*n* = 15)	NAC (*n* = 113)	5 y: 74%	5 y: 61%	Deaths (*n* = 3) ETC (*n* = 13)
Goorin et al. [10]	2003	Prospective	100	<12 (*n* = 19) >12 (*n* = 26)	-	Femur (*n* = 26) Tibia (*n* = 11)	NAC (*n* = 45)	5 y: 76%	5 y: 61%	Deaths (*n* = 3) [CHF (*n* = 2) BO (*n* = 1)] SHL (*n* = 1)
				<12 (*n* = 23) >12 (*n* = 32)		Femur (*n* = 35) Tibia (*n* = 13)	ACT (*n* = 55)	5 y: 79%	5 y: 69%	
Zalupski et al. [16]	2004	Prospective	63	22 (12–70)	79	Femur (*n* = 32) Tibia (*n* = 16)	NAC (*n* = 63)	5 y: 58%	NR	Death (*n* = 1) [SML (*n* = 1)] ETC (*n* = 11)
Ford et al. [17]	2004	Retrospective	428	15.8	-	Femur (*n* = 271) Tibia (*n* = 157)	NAC (*n* = 428)	-	-	NR
			Center 1 (*n* = 265)	15.2		Femur (*n* = 171) Tibia (*n* = 94)		5 y: 73%	5 y: 56%	
			Center 2 (*n* = 163)	16.1		Femur (*n* = 100) Tibia (*n* = 63)		5 y: 60%	5 y: 43%	
Wilkins et al. [18]	2005	Prospective	62	14 (5–21)	91	Femur (*n* = 40) Tibia (*n* = 14)	NAC (*n* = 62)	5 y: 96.6% 10 y: 93.2%	5 y: 86.4% 10 y: 86.4%	Deaths (*n* = 0) G3-Ct (*n* = 1) G3-OT (*n* = 1)
Bacci et al. [19]	2005	Retrospective	1148	16.7 (3–40)	177.6	Femur (*n* = 612) Tibia (*n* = 327) Humerus (*n* = 128)	NAC (*n* = 900)	5 y: 66% (1148) 10 y: 57% (1148)	5 y: 61%	Death from Toxicity (*n* = 12)
							ACT (*n* = 248)		5 y: 43%	Death from Toxicity (*n* = 6)
Ferrari et al. [20]	2005	Prospective	182	16 (4–40)	55	Femur (*n* = 93) Tibia (*n* = 48) Humerus (*n* = 30)	NAC (*n* = 182)	5 y: 77%	5 y: 64%	Death from Toxicity (*n* = 3)
Petrilli et al. [21]	2006	Prospective	Included in This Review: 168 out of 209	≤14 (*n* = 90) >14 (*n* = 88)	61	Femur (*n* = 96) Tibia (*n* = 58)	NAC (*n* = 168)	5 y: 60.5% 10 y: 55.2%	5 y: 45.5% 10 y: 45.5%	Deaths from Toxicity (*n* = 15 out of 209)
Basaran et al. [22]	2007	Prospective	38	22 (15–41)	64	Tibia (*n* = 16) Femur (*n* = 14) Humerus (*n* = 6)	NAC (*n* = 38)	5 y: 48.2%	5 y: 41.9%	Severe HT (*n* = 12) Severe Nausea (*n* = 2) Severe Vomiting (*n*= 2) Severe FN (*n* = 9)
Lewis et al. [23]	2007	Prospective	497	15 (12–18)	62	Femur (*n* = 296) Tibia (*n* = 116)	NAC (*n* = 497)	-	-	G3/G4 Toxicity (*n* = 468)
							Reg. C (*n* = 245)	5 y: 55%	5 y: 39%	Excessive Toxicity (*n* = 12)
							Reg. DI (*n* = 252)	5 y: 58%	5 y: 41%	Excessive Toxicity (*n* = 17)
Iwamoto et al. [24]	2009	Prospective	113	15 (6–27)	75.6	Femur (*n* = 62) Tibia (*n* = 33)	NAC (*n* = 113)	5 y: 77.9%	5 y: 65.5%	Deaths from Toxicity (*n* = 6)
Bielack et al. [25]	2009	Retrospective	Included in This Review: 2017 out of 2464	15.4	67.92	(Out of 2464) Femur (*n* = 1229) Tibia (*n* = 636)	NAC (*n* = 2017)	5 y: 74.8% 10 y: 69.5%	NR	(Out of 2464) Deaths from Toxicity during Primary Treatment (*n* = 29) Secondary Malignancy (*n* = 12) Cardiomyopathy (*n* = 7)
Hong et al. [26]	2011	Retrospective	124	<15 (*n* = 36) 15–40 (*n* = 82) >40 (*n* = 6)	68.4	DF (*n* = 68) PT (*n* = 24) PH (*n* = 16)	AP NAC (*n* = 77) IAP NAC (*n* = 47)	5 y: 85% 5 y: 63.9%	5 y: 78.7% 5 y: 63.6%	Deaths from Toxicity: (*n* = 5) [during Adjuvant Chemotherapy]
					46.8					
Smeland et al. [27]	2011	Prospective	63	15 (8–39)	77	Femur (*n* = 34) Tibia (*n* = 15)	NAC (*n* = 63)	5 y: 76%	5 y: 65%	Deaths from Toxicity (*n* = 3) NE (*n* = 1) G4 Ct (*n* = 1)
Li et al. [28]	2011	Retrospective	111	18 (14–39)	28	DF (*n* = 49) PT (*n* = 32)	NAC (*n* = 111)	3 y: 68.3%	3 y: 65.9%	Deaths from Toxicity (*n* = 0)
							ST NAC (*n* = 87)	3 y: 80.0%	3 y: 79.5%	
							NST NAC (*n* = 24)	3 y: 38.9%	3 y: 30.1%	
Whelan et al. [29]	2012	Retrospective	1067	15 (3–40)	112.8	Femur (*n* = 611) Tibia (*n* = 264)	NAC (*n* = 1067)	5 y: 56% 10 y: 52%	5 y: 43% 10 y: 42%	NR
Kudawara et al. [30]	2013	Retrospective	40	0–15 (*n* = 17) 16–20 (*n* = 16) 21–30 (*n* = 2) >30 (*n* = 5)	117	Femur (*n* = 20) Tibia (*n* = 13)	NAC (*n* = 40)	5 y: 98% 10 y: 95%	5 y: 83% 10 y: 80%	Deaths from Toxicity (*n* = 0) G4 Lp (52% of cycles) G4NphT (*n* = 1)
Xu et al. [31]	2014	Retrospective	39	16 (6–39)	66	DF (*n* = 21) PT (*n* = 13)	NAC (*n* = 39)	5 y: 61.5%	5 y: 54.8%	Deaths from Toxicity (*n* = 0)
Ferrari et al. [32]	2014	Retrospective	171	16 (3–40)	39	Femur (*n* = 92) Tibia (*n* = 56)	NAC (*n* = 171)	5 y: 81%	5 y: 50%	G4Lp (*n* = 171) G4Thr (*n* = 58)
Bajpai et al. [33]	2017	Retrospective	Included in This Review: 209 out of 237	(209) 17 (6–56)	(209) 35.6	(237) Femur (*n* = 109) Tibia (*n* = 83)	NAC (*n* = 209)	3 y: 82% 5 y: 80%	3 y: 63% 5 y: 60%	Deaths from Toxicity (*n* = 2)
Huang et al. [34]	2018	Retrospective	69	20 (12–57)	75.9	Proximal Tibia Only	NAC (*n* = 69)	3 y: 91.3% 5 y: 87%	-	NR
Morsy et al. [35]	2019	Retrospective	30	5–9 (*n* = 5) 10–14 (*n* = 13) 15–18 (*n* = 12)	63	DF (*n* = 15) PT (*n* = 6)	NAC (*n* = 30)	3 y: 79% 5 y: 65.3%	3 y: 69.5% 5 y: 65.2%	NR

Key—OS: overall survival, DFS: disease-free survival, EFS: event-free survival, TR: treatment related, DF: dital femur, PT: proximal tibia, PH: proximal humerus, PF: proximal femur; NAC: neoadjuvant chemotherapy (+ surgery + adjuvant chemotherapy), ACT: adjuvant chemotherapy only, y: years, m: months, NR: not reported; ETC: early termination of chemotherapy, CHF: congestive heart failure, BO: bronchiolitis obliterans, SHL: severe hearing loss; SML: severe myelossupression, G3: grade three (severe toxicity), Ct: cardiotoxicity, OT: ototoxicity, Mc: mucositis; Reg. C: core regimen, Reg. DI: dose-intensity regimen, G4: grade four (life-threatening toxicity), HT: hematological toxicity, FN: febrile neutropenia; AP: double therapy regimen; IAP: triple therapy regimen, NE: necrotizing enterocolitis; Lp: leukopenia, ST: standard, NST: non-standard, NphT: nephrotoxicity. * In this study, “survival refers to those patients who were disease-free at last follow up”, a similar definition to that used as overall-survival in other studies.

**Table 3:** Summary of outcomes and side-by-side comparison of NAC and ACT strategies among the studies selected for this review.

Total Number of Patients Involved		5-Year Overall-Survival Range		5-Year Disease-Free Survival/ Event-Free Survival Range		Treatment-Related Toxicity	
NAC	ACT	NAC	ACT	NAC	ACT	NAC	ACT
6789	364	55–80%	77–79%	39–65.5%	43–69%	Early Termination/Modification of Chemotherapy: 125 patients (≈1.84%) Reported Deaths Directly Attributed to Chemotherapy: 25 (≈0.37%)	Reported Deaths Directly Attributed to Chemotherapy: 7 (≈1.92%)
